# Muscle Mass and Muscle Strength in Non-Dialysis-Dependent Chronic Kidney Disease Patients

**DOI:** 10.3390/jcm13216448

**Published:** 2024-10-28

**Authors:** Katarzyna Romejko, Katarzyna Szamotulska, Aleksandra Rymarz, Stanisław Niemczyk

**Affiliations:** 1 Department of Internal Diseases, Nephrology and Dialysis, Military Institute of Medicine–National Research Institute, 128 Szaserów Street, 04-141 Warsaw, Poland; arymarz@wim.mil.pl (A.R.); sniemczyk@wim.mil.pl (S.N.); 2 Department of Epidemiology and Biostatistics, Institute of Mother and Child, 17a Kasprzaka Street, 01-211 Warsaw, Poland; katarzyna.szamotulska@imid.med.pl

**Keywords:** chronic kidney disease, sarcopenia, skeletal muscle mass, muscle strength, anthropometric variables, clinical variables

## Abstract

**Background:** Sarcopenia is a state with a progressive and generalized loss of skeletal muscle mass and strength. However, muscle strength and muscle mass are different features, which are usually not studied separately. The aim of the study was to investigate anthropometric and clinical correlates and sources of variation in both skeletal muscle mass and muscle strength in chronic kidney disease (CKD). **Methods:** The study sample consisted of 84 patients with an estimated glomerular filtration rate (eGFR) < 45 mL/min/1.73 m^2^. Muscle strength was estimated by measuring hand grip strength (HGS). Muscle quantity was measured with bioimpedance spectroscopy (BIS). Serum creatinine, urea, uric acid (UA), and albumin were measured as well. **Results:** Appendicular skeletal muscle mass (ASM) significantly and positively correlated with body mass, NH weight (normally hydrated weight), height, body mass index (BMI), lean tissue mass (LTM), lean tissue index (LTI), fat mass (FM), and fat tissue index (FTI), and was negatively associated with hydration status. HGS significantly and positively correlated with body mass, NH weight, height, LTM, LTI, and ASM, and was negatively associated with UA and urea. After adjustment for age, sex, and height, HGS remained significantly and negatively related with UA and hydration status. **Conclusions:** In CKD patients, ASM is determined by anthropometric parameters, but HGS is determined by both anthropometric and clinical variables specific for CKD. In order to study the determinants of HGS in CKD, relationships with HGS should be adjusted for anthropometric variables.

## 1. Introduction

Low muscle strength and low skeletal muscle mass or poor skeletal muscle quality, observed simultaneously, are the main criteria for the diagnosis of sarcopenia [1]. Sarcopenia is mainly associated with the process of aging; however, it also occurs with a sedentary lifestyle and numerous pathological states such as heart failure, diabetes, chronic kidney disease (CKD), acute or chronic inflammation, and both malnutrition and obesity [2]. Low skeletal muscle mass and strength are associated with high susceptibility to injuries, increased morbidity, higher numbers of hospitalizations, and elevated mortality [3,4]. Additionally, sarcopenia significantly reduces quality of life [5].

CKD is one of the major public health problems [6]. Almost 13% of the world’s population suffers from irreversible kidney damage. The prevalence of sarcopenia in CKD patients is up to 55% [7]. The causes of sarcopenia in CKD are multivariate, including low-grade inflammatory state, metabolic acidosis, insulin resistance, hyperparathyroidism, resistance to growth hormone, derangements of adipocytokine profile, and hypogonadism [8]. However, the studies that examine sarcopenia in CKD patients are not numerous, and there is a need to expand the knowledge on this topic [9].

The diagnostic criteria for sarcopenia are based on the updated guidelines of European Working Group on Sarcopenia in Older People (EWGSOP2) 2018 [1]. They are also used in research studies on CKD; however, there is still a lack of specific assessment guidelines for patients with kidney function decrease [10]. The revised European consensus on the definition and diagnosis of sarcopenia in 2018 confirmed that impaired muscle strength is the most reliable tool to assess muscle function. Therefore, if low muscle strength is diagnosed, sarcopenia is probable. Sarcopenia is confirmed with the presence of low skeletal muscle quantity or quality and is severe when low physical performance also occurs [1]. This way, muscle strength and muscle mass are usually studied together [11,12]. However, muscle strength and muscle mass are different features, which is rarely noticed [13,14]. In CKD patients, understanding the associations between muscle strength and muscle mass separately, with clinical and anthropometric parameters that are easily available in everyday medical practice, may improve the effectiveness of sarcopenia prevention. Moreover, there are patients with low muscle strength but correct muscle quality or quantity, and conversely, with normal muscle strength and decreased muscle mass. These patients cannot be diagnosed as sarcopenic, and this situation does not allow the implementation of preventive and therapeutic methods to slow down the loss of muscle strength or muscle mass [15,16,17].

There are several methods to evaluate muscle strength and muscle quantity. Muscle strength may be evaluated with the use of a hand grip strength (HGS) test or chair stand test. The measurement of HGS requires the use of a calibrated dynamometer. It has been found that HGS correlates with strength in other body compartments and may play a role as a surrogate for more accurate measurements of arm and leg strength [18]. As this method is simple, it may be widely available in hospitals or outpatient clinics. The cut-off points for low muscle strength measured with HGS are <27 kg for men and <16 kg for women [1].

Several techniques, such as magnetic resonance imaging (MRI), computed tomography (CT), and dual-energy X-ray absorptiometry (DXA), evaluate skeletal muscle mass [1]. Another method that estimates skeletal muscle mass is bioelectrical impedance analysis (BIA). It is a simple, inexpensive, and easy to use method, and the equipment is portable so it does not require patient transport. Moreover, BIA does not require patient exposure to X-rays. Single-frequency BIA uses a current of 50 kHz, and by measuring the whole-body electrical conductivity, estimates total body water, fat-free mass (FFM), and fat mass (FM). The main disadvantage of this method is that overhydration may overestimate lean body mass; this is why the method that measures body composition more accurately is multifrequency bioimpedance spectroscopy (BIS) [19,20]. BIS uses multiple current frequencies (1–1000 kHz) and provides a more precise determination of total body water (TBW), distinguishing between extracellular water (ECW) and intracellular water (ICW) and lean tissue mass (LTM) and adipose tissue mass (ATM). There are numerous equations to evaluate appendicular skeletal muscle mass (ASM) based on bioimpedance analysis in different groups of people [21,22,23].

The aim of the study is to investigate anthropometric and clinical correlates and sources of variation in skeletal muscle mass and muscle strength in CKD patients. The main analysis was preceded by validation of Lin’s estimation of ASM by bioimpedance spectroscopy in Polish CKD patients [22].

## 2. Materials and Methods

### 2.1. Participants and Eligibility Criteria

We performed a cross-sectional study that included patients with an estimated glomerular filtration rate (eGFR) < 45 mL/min/1.73 m^2^. Patients with CKD, not treated with dialysis, who were qualified for the study visited the Nephrological Outpatient Clinic of the Military Institute of Medicine—National Research Institute in Warsaw, Poland, for a routine check-up. The inclusion criteria were age between 18 and 90 years and eGFR < 45 mL/min/1.73 m^2^. The exclusion criteria were clinical signs of infection, the presence of metal parts in the body, and renal replacement therapy or its requirement within the following 3 months. Each participant signed an informed consent.

### 2.2. Laboratory Measurements

Blood samples for laboratory measurements such as creatinine, urea, uric acid (UA), and albumin were taken after an overnight fast and were analyzed in the local department of laboratory diagnostics. Serum creatinine concentrations were analyzed using the Jaffe method (Gen.2, Roche Diagnostics GmbH, Rotkreuz, Switzerland), serum urea levels using the urease kinetic test (Cobas c501, Roche Diagnostics, GmbH, Rotkreuz, Switzerland), and serum UA with the use of the enzymatic colorimetric test (Cobas c501, Roche Diagnostics, GmbH, Rotkreuz, Switzerland). Plasma albumin levels were measured with the use of the BCP Albumin Assay Kit (Roche Diagnostics GmbH, Rotkreuz, Switzerland).

### 2.3. The Assessment of eGFR

eGFR (mL/min per 1.73 m^2^) was calculated according to the short modification of diet in renal disease (MDRD) formula [24,25]:

$$eGFR=175\times {SerumCr}^{-1.154}\times {age}^{-0.203}\times 1.212\;(if\;patient\;is\;Black)\times 0.742\;(if\;female)$$

### 2.4. The Diagnosis of Diabetes Mellitus Type 2

Diabetes mellitus type 2 was diagnosed based on the patient’s medical history and the results of an oral glucose tolerance test (OGTT) with the value of serum glucose concentration ≥ 200 mg/dL after 120 min of 75 g oral glucose load [26].

### 2.5. The Assessment of Muscle Strength

Muscle strength was estimated by measuring HGS with the use of a handheld hydraulic dynamometer (Saehan Corporation, Changwon, Republic of Korea). Subjects were asked to stay in a seated position, with the elbow flexed at a 90° angle, the forearm and wrist in a neutral position, and to grip the dynamometer with both dominant and non-dominant hand as hard as possible three times for intervals lasting 30 s. Then, the arithmetic mean for each hand was measured. The final result for HGS was the arithmetic mean of the measurements for both hands.

### 2.6. The Assessment of Skeletal Muscle Mass

Muscle quantity was measured with BIS using a body composition monitor (BCM, Fresenius Medical Care, Bad Homburg, Germany). Patients were asked to stay in the supine position after a five-minute rest with electrodes placed on one hand and one foot in a tetrapolar configuration. In order to evaluate muscle mass from BCM, we used Lin’s algorithm, which derives a formula for ASM estimation (kg) based on parameters obtained from bioimpedance spectroscopy and the sum of fat-free soft tissues in the arms and legs assessed from DXA [22]:

ASM_BCM_ = −1.838 + 0.395 × TBW + 0.105 × body weight − 0.026 × age + 1.231 (if male)

Lin applied his prediction model to the validation group of 108 CKD Taiwanese patients, obtaining a Pearson correlation coefficient r = 0.953 between estimated ASM_BCM_ and ASM_DXA_ (*p* < 0.001). The limit of agreement in Bland–Altman analysis of model-derived ASM compared with DXA-derived ASM was 0.098 ± 2.440 kg.

In order to validate Lin’s prediction model in Polish CKD patients, we applied Lin’s formula to the independent sample of 109 CKD patients (eGFR < 30 mL/min/1.73 m^2^) in which both DXA and BCM measurements were collected. This study design has been previously described [27]. The Pearson correlation coefficient between estimated ASM_BCM_ and ASM_DXA_ was 0.954 (*p* < 0.001). The limit of agreement in Bland–Altman analysis of model-derived ASM compared with DXA-derived ASM was 0.949 ± 2.698 kg.

### 2.7. Statistical Analysis

The results are presented as means ± standard deviations (SDs) and proportions. Bivariate associations between continuous variables were assessed by Pearson correlation coefficient; between continuous and categorical variables by Student’s t-test or analysis of variance, depending on the number of categories; and between categorical variables by the chi-square test. For multivariate analysis, stepwise forward linear regression was applied. A *p* value < 0.05 was considered to be statistically significant. Statistical analysis was performed using IBM SPSS Statistics for Windows, Version 25.0. Armonk, NY, USA: IBM Corp.

## 3. Results

The study sample consisted of 84 patients, 38 women (45.2%) and 46 men (54.8%), with eGFR < 45 mL/min/1.73 m^2^: 22 patients (26.2%) with moderately to severely decreased eGFR (30–44 mL/min/1.73 m^2^—stage G3b of CKD), 55 patients (65.5%) with severely decreased eGFR (15–29 mL/min/1.73 m^2^—stage G4 of CKD), and 7 individuals (8.3%) with non-dialysis kidney failure (eGFR < 15 mL/min/1.73 m^2^—stage G5 of CKD) [28]. The mean age of the patients was 68.7 ± 14.6 years, the range was 24–90 years, and 86.9% of the patients were overweight or obese: 41.7% of participants had a body mass index (BMI) 25.0–29.9 kg/m^2^ and 45.2% of patients had BMI ≥ 30.0 kg/m^2^.

Men had higher HGS and higher ASM compared with women (*p* < 0.001, *p* < 0.001). Men were taller and heavier than women (*p* < 0.001, *p* = 0.018). The LTM, lean tissue index (LTI), and relative fat mass (RFM) significantly differed between men and women, with higher LTM and LTI in men (*p* < 0.001, *p* = 0.037) but increased RFM in women (*p* = 0.010) (Table 1).

We found that 19.5% of participants were anemic, with serum hemoglobin concentrations below 11.0 g/dL. The majority of patients, 88.9%, had plasma albumin concentrations within the normal range, and 11.1% of individuals had decreased serum albumin. Furthermore, 62.3% of patients showed elevated plasma (UA). Overhydration (OH) was observed in 33.3% of patients. Diabetes mellitus was diagnosed in 25.0% of individuals (Table 2).

We found statistically significant strong or moderate correlations between ASM and almost all the studied anthropometric variables in both sexes. ASM was strongly increasing with higher body mass, NH weight (normally hydrated weight) and BMI (*p* < 0.001, *p* < 0.001, *p* < 0.001, respectively). Height was positively correlated with ASM: taller participants had elevated ASM (*p* < 0.001 for women, *p* = 0.004 for men). We observed significant positive correlations between ASM and LTM (*p* = 0.001 for women, *p* < 0.001 for men) but found that LTI was positively associated with ASM in the male population only (*p* < 0.001). Moreover, ASM increased with the rise of FM and fat tissue index (FTI) in both sexes (*p* < 0.001, *p* < 0.001, respectively), but the correlations were stronger for women. ASM was associated significantly with RFM only in the group of women (*p* = 0.034), but moderately (Table 3).

We also found statistically significant moderate associations between HGS and anthropometric measurements in the studied population. HGS was positively correlated with body mass and NH weight in women (*p* = 0.003, *p* = 0.005, respectively). In the group of women, those with increased BMI and FM had also higher HGS (*p* = 0.003, *p* = 0.015, respectively). There was a statistically significant relationship between HGS and height. Taller individuals had higher HGS in both sexes (*p* < 0.001 for women, *p* = 0.002 for men). Additionally, HGS was positively associated with LTM in men (*p* = 0.018). The positive significant relationship was also observed between HGS and ASM in women (*p* = 0.001) (Table 3).

Both ASM and HGS negatively correlated with age. The Pearson correlation coefficient for association with ASM was r = −0.555 for men (*p* < 0.001) and r = −0.288 for women (*p* = 0.080), and that for association with HGS was r= −0.459 for women (*p* = 0.004) and r = −0.608 for men (*p* < 0.001).

Table 4 presents the correlations between ASM, HGS, and clinical variables. There was a statistically significant relationship between ASM and certain clinical parameters. Women with higher serum albumin showed lower ASM (*p* = 0.011). We also found that participants with increased OH had lower ASM in both sexes (*p* = 0.006 in women, *p* = 0.026 in men). Men with diabetes had higher ASM compared with those without diabetes (*p* = 0.014).

Additionally, we observed a statistically significant relationship between HGS and clinical variables in the studied population. HGS was much lower in male participants with serum urea above normal values (*p* < 0.003); however, all female participants presented concentrations of serum urea above the upper limit of normal (ULN). Additionally, individuals with serum uric acid above the normal range also had lower HGS (*p* < 0.039 for both sexes together) (Table 4).

Finally, stepwise forward multiple linear regression was applied to investigate the relationship of HGS with the clinical variables in multivariate analysis. Clinical variables significantly associated with HGS or ASM in univariate analysis (UA, OH, urea) were considered for estimation of the final model together with non-modifiable variables such as gender, age, and height. In the estimated model, height, gender, age, serum concentration of UA, and absolute overhydration remained statistically significant (Table 5). These variables accounted for 62.1% of the statistical variability in HGS.

## 4. Discussion

In our study, we evaluated whether the chosen anthropometric and clinical variables were related, to the same extent, to the components of sarcopenia—skeletal muscle mass and muscle strength—in the population of patients with eGFR < 45 mL/min/1.73 m^2^.

### 4.1. Appendicular Skeletal Muscle Mass and Anthropometric Variables

ASM was significantly associated with anthropometric variables. ASM was positively correlated with total body mass, NH weight, height, BMI, LTM, and fat in both sexes. However, LTI was correlated with ASM only in the group of men, and RFM only in the female population (Table 3). In our study, height was strongly associated with ASM, which may depend on the greater total mass of skeletal muscles in taller patients. These relationships are compatible with many existing predictive studies, in which the ASM estimating equations usually include body weight or normalize ASM for height [29,30].

BMI and total FM were also positively associated with ASM. Increased ASM in individuals with higher total body mass or BMI may be due to higher absolute muscle mass, as neither total body mass nor BMI allow the differentiation of muscle or FM. According to the reverse epidemiology phenomenon in CKD, which states that obese patients have lower morbidity and higher survival rates compared with malnourished individuals, and knowing that higher skeletal muscle mass is correlated with decreased mortality, based on our findings, the obesity paradox in CKD may be the result of higher ASM in obese subjects [31,32]. Tomborelli Bellafronte also presumed that the obesity paradox in the CKD population may be the result of the protective effect of lean mass, even in patients with greater adiposity [33]. We may conclude that taller and heavier patients with CKD have higher ASM. However, regardless of the associations between anthropometric variables and ASM, we need to be aware that all these variables are correlated. This means that each single studied anthropometric parameter includes information about every other one. Therefore, it is difficult, if not impossible, to distinguish which anthropometric parameters are crucial for ASM, especially in cross-sectional designs.

### 4.2. Hand Grip Strength and Anthropometric Variables

In our study, we found that HGS was associated with anthropometric parameters such as total body mass, NH weight, height, LTM, LTI, and ASM. The statistical significance in the group of women was observed between HGS and body mass, NH weight, height, BMI, fat, and ASM. In the group of men, only the relationships between HGS and height and between HGS and LTM remained statistically significant (Table 3). There are numerous studies that confirm the relationship between HGS and certain anthropometric variables in the general population. The study of Cheng, which included 10,407 participants, reported that patients with higher BMI also had increased HGS [34]. Very interesting also are the results of Curtis, who observed that lower BMI was correlated with higher likelihood of probable sarcopenia (defined as decreased muscle strength), whereas overweight and obesity seemed to play a protective role [35]. HGS was also observed to be associated with FM. There are studies that found negative relationships between HGS and total FM as well as between HGS and RFM [36,37]. In CKD patients, individuals with increased total body fat had lower HGS, according to Jiang [36]. We obtained opposite results, but only in the group of women—participants with elevated total FM showed higher HGS (*p* = 0.015).

Regarding height, this was the only anthropometric parameter that was positively correlated with HGS in our study, both when data were mixed and when data were separated. In multivariate analysis, we also observed that height remained a significant determinant of HGS in the group of CKD patients (*p* < 0.001) (Table 3 and Table 5). There are many studies that confirm the positive association between HGS and height or weight in the general population [38,39,40,41]. The number of studies that evaluate the anthropometric determinants of HGS in the CKD population is small. The study of Corona, which included patients with dialysis kidney failure, found that HGS was associated with both height and weight [42]. Hasheminejad also found a significant positive relationship between HGS and height and between HGS and weight in kidney failure individuals [43]. We observed a positive correlation between HGS and weight in the female population only (*p* = 0.005).

### 4.3. Appendicular Skeletal Muscle Mass and Clinical Variables

We found that ASM was significantly negatively associated with serum albumin in women; those with plasma albumin within the normal range presented lower ASM compared with participants with decreased albumin, who had higher ASM (*p* = 0.011). Serum albumin is known to play a role as a nutritional marker. Decreased serum albumin is one of the diagnostic criteria of protein-energy wasting (PEW) in CKD, which includes also the degree of muscle mass loss [44]. The probable reason for our results is confounding by age. The mean age of women with correct serum albumin in our study was 68 years, and in women with decreased serum albumin, it was significantly lower—45 years. Since age negatively correlates with ASM, this is the most likely reason why women with higher serum albumin had lower ASM in our study. The study of Chen found similar results to ours in the group of women—they also observed a negative association between muscle mass and serum albumin, but in the group of men, this relationship was positive [44,45]. Additionally, there are studies that have reported that serum albumin is a poor nutritional marker in patients with dialysis kidney failure [46].

We also found that ASM was associated with OH and relative overhydration (ROH). Participants with higher absolute OH and ROH had lower ASM when the data of men and women were mixed (*p* < 0.001, *p* = 0.003, respectively). Absolute OH was also higher in patients with lower ASM when the data were separated (*p* = 0.006 for women, *p* = 0.026 for men). In relation to ROH, when the data were separated, we observed such an association in the female population only (*p* = 0.005) (Table 4). Our results are in opposition to the results of other studies, in which skeletal muscle mass was positively associated with hydration status [47]. A possible reason for the positive association between fluid overload and ASM may be that overhydration overestimates the mass of skeletal muscle [47]. Additionally, the assessment of hydration status may depend on the technique used to evaluate body composition [48]. In our study, the negative effect of excessive fluid load on ASM may be caused by the fact that patients with OH had, on average, much lower BMI than other participants (Table 4).

We observed that men with diabetes had higher ASM (*p* = 0.014), which is in opposition to some other studies (Table 4). The report of Hong, which included over 200,000 participants in a cohort study, found that skeletal muscle mass was negatively associated with the development of diabetes mellitus [49]. Larsen, also using a multivariate approach, observed that in the group of women with normal body weight, lower skeletal muscle mass was associated with a higher incidence of diabetes [50]. The different results of our study may be related to the higher body mass of patients with diabetes compared with non-diabetic individuals in our sample (Table 4). Also, muscle mass loss in the diabetic state may be affected by numerous factors, and not all patients with diabetes mellitus have decreased muscle mass [51].

### 4.4. HGS and Clinical Variables

In our study, we found a negative association between HGS and UA, also in multivariate analysis after adjusting for age, gender, and height (*p* = 0.041) (Table 5). Participants with lower plasma UAs had higher HGSs. In several studies performed in the general population, the relationship between UA and HGS was the subject of observation, and the conclusions were not clear [52,53,54]. The association between UA and muscle strength may be due to the role of UA in oxidative balance. The increased subclinical inflammatory state in CKD is known to be a significant factor contributing to PEW and sarcopenia. UA may have both pro-oxidative and anti-oxidative properties, depending on its extracellular or intracellular actions [55]. In our study, higher serum UA was associated with lower HGS in CKD patients, which may be associated with increased pro-inflammatory properties of UA in the studied population.

Since OH was related to ASM in bivariate analysis, we decided to include OH in the multivariate model for HGS and obtained a significant negative association with HGS (*p* = 0.042) after adjusting for age, gender, and height (Table 5). Patients with fluid overload showed lower HGS; therefore, it may be concluded that OH can contribute to the development of sarcopenia in CKD. There are not many reports on the relationship between OH and HGS. Garagarza conducted a study with 155 dialysis kidney failure patients and also observed a negative relationship between HGS and OH [56]. Future studies on the impact of fluid overload on muscle strength in CKD patients are needed.

We noticed a significant bivariate relationship between HGS and serum urea. Patients with plasma urea concentrations within the normal range had significantly higher HGS compared with individuals with elevated serum urea levels (*p* < 0.001). Urea belongs to uremic toxins, increasing with the deterioration of kidney function. In our study, only three participants had correct serum urea concentrations, which is an expected result for the population with kidney function decrease. The small number of participants with correct serum urea concentrations caused us to exclude urea from the final regression model after implementing the stepwise procedure (Table 5).

We did not observe an association between HGS and hemoglobin. Anemia is known to be related to weaker muscle strength. It is probably caused by impaired muscular oxygen transport and consumption, which leads to chronic tissue hypoxia and worsens muscular function. The results of numerous studies in the general population that examined the relationship between hemoglobin and HGS are not consistent [57,58,59]. Despite the fact that patients with CKD are prone to develop anemia, our study did not show a relationship between hemoglobin and HGS in either the male or female population.

HGS may also depend on diabetic status. The mechanisms of muscle strength decrease due to hyperglycemia are multifactorial, including insulin resistance, increased secretion of inflammatory cytokines, and oxidative stress. There are numerous studies that evaluated the association between HGS and hyperglycemia in the general population [60,61]. In our study of CKD patients, we found no relationship between diabetes and HGS.

We also did not observe a significant relationship between HGS and serum albumin. We observed that in the group of men, higher albumin concentrations were accompanied by higher HGS, contrary to women, in whom those with serum albumin levels within the normal range had lower HGS. The probable reason for our results is confounding by age, as explained above, in the paragraph on the relationship between ASM and serum albumin.

One of the limitations of our study is that a non-traceable method to measure creatinine was employed (Jaffe) and an old estimated glomerular filtration rate (not measured) formula was used (MDRD). Another limitation of our study is its relatively small sample size. A larger number of participants would enable a more detailed and more comprehensive analysis. Additionally, a cohort design would be more appropriate than a cross-sectional study and would allow the establishment of initial conditions. Probably another limitation of our study is that HGS was measured as the arithmetic mean of two arms, which is not the most common approach but seemed to be more repeatable. The strength of our study is that it was performed in the CKD population, for which the knowledge of the determinants of decreased ASM, HGS, and sarcopenia is limited. Also, our study takes into account gender, which reflects the different anthropometric characteristics in men and women.

## 5. Conclusions

In summary, our study confirms that muscle strength and muscle mass are different features and may depend on different parameters. According to the findings of our study, which evaluated separately the determinants of ASM and HGS in CKD patients, we may conclude that, apart from the negative correlation with age, ASM is associated with anthropometric parameters, but HGS is correlated with both anthropometric and clinical variables that are specific to CKD advancement. It seems that in order to study the determinants of HGS in CKD, relationships with HGS should be adjusted for anthropometric variables.

## Figures and Tables

**Table 1:** Anthropometric characteristics of the studied population.

Anthropometric Variables	Total		Women		Men		P_men vs. women_
	n	Mean ± SD	n	Mean ± SD	n	Mean ± SD	
HGS (kg)	84	25.27 ± 10.09	38	18.88 ± 5.31	46	30.55 ± 10.07	**<0.001**
ASM (BIS)	84	19.72 ± 4.89	38	16.90 ± 4.42	46	22.04 ± 3.97	**<0.001**
Age (years)	84	68.71 ± 14.61	38	66.53 ± 15.27	46	70.52 ± 13.94	0.214
Body mass (kg)	84	82.87 ± 18.88	38	77.58 ± 20.57	46	87.25 ± 16.33	**0.018**
NH weight (kg)	84	82.20 ± 19.04	38	77.54 ± 21.10	46	86.04 ± 16.40	**0.041**
Height (cm)	84	164.65 ± 8.57	38	158.11 ± 6.86	46	170.07 ± 5.54	**<0.001**
BMI (kg/m^2^)	84	30.45 ± 6.02	38	30.74 ± 6.66	46	30.21 ± 5.49	0.685
LTM (kg)	84	33.39 ± 8.71	38	28.80 ± 7.61	46	37.18 ± 7.74	**<0.001**
LTI	84	12.26 ± 2.91	38	11.53 ± 3.19	46	12.87 ± 2.55	**0.037**
Fat (kg)	84	35.24 ± 13.17	38	35.42 ± 14.93	46	35.09 ± 11.69	0.909
RFM (%)	84	41.64 ± 8.53	38	44.24 ± 9.52	46	39.49 ± 7.00	**0.010**
FTI	84	17.65 ± 6.40	38	19.02 ± 7.18	46	16.53 ± 5.50	0.075

HGS, hand grip strength; ASM (BIS), appendicular skeletal muscle mass measured with bioimpedance spectroscopy; NH weight, normally hydrated weight; BMI, body mass index; LTM, lean tissue mass; LTI, lean tissue index; RFM, relative fat mass; FTI, fat tissue index; *p*-values < 0.05 are marked in bold.

**Table 2:** Clinical characteristics of the studied population.

Clinical Variables	Total		Women		Men		P_men vs. women_
	n	%	n	%	n	%	
Serum albumin (g/dL)							
<3.9	9	11.1%	3	8.3%	6	13.3%	0.722
3.9–4.9	72	88.9%	33	91.7%	39	86.7%	
Hemoglobin (g/dL)							
<11.0	16	19.5%	8	21.1%	8	18.2%	0.744
11.0–18.0	66	80.5%	30	78.9%	36	81.8%	
eGFR (mL/min/1.73 m^2^)							
≤29	62	73.8%	28	73.7%	34	73.9%	0.981
30–44	22	26.2%	10	26.3%	12	26.1%	
Serum creatinine (xULN)							
<1.5	18	21.4%	15	39.5%	3	6.5%	**0.001**
1.5–2.0	31	36.9%	14	36.8%	17	37.0%	
2.0–3.0	27	32.1%	6	15.8%	21	45.7%	
>3.0	8	9.5%	3	7.9%	5	10.9%	
Serum urea (xULN)							**0.012**
≤1	3	4.2%	0	0.0%	3	7.7%	
1–1.5	20	27.8%	11	33.3%	9	23.1%	
1.5–2	22	30.6%	15	45.5%	7	17.9%	
2–2.5	16	22.2%	3	9.1%	13	33.3%	
>2.5	11	15.3%	4	12.1%	7	17.9%	
Serum uric acid (mg/dL)							**0.032**
W: 2.4–5.7, M: 3.4–7.0	29	37.7%	9	25.0%	20	48.8%	
W: >5.7, M: >7.0	48	62.3%	27	75.0%	21	51.2%	
OH (L)							
<−1.0	9	10.7%	4	10.5%	5	10.9%	**0.006**
−1.0–1.0	47	56.0%	28	73.7%	19	41.3%	
>1.0	28	33.3%	6	15.8%	22	47.8%	
ROH (%)							
<−7.0	8	9.5%	4	10.5%	4	8.7%	**0.017**
−7.0–7.0	52	61.9%	29	76.3%	23	50.0%	
>7.0	24	28.6%	5	13.2%	19	41.3%	
Diabetes mellitus type 2							
No	63	75.0%	28	73.7%	35	76.1%	0.800
Yes	21	25.0%	10	26.3%	11	23.9%	

eGFR, estimated glomerular filtration rate; ULN, upper limit of normal; W, women; M, men; OH, overhydration; ROH, relative overhydration; *p*-values < 0.05 are marked in bold.

**Table 3:** The associations between appendicular skeletal muscle mass and hand grip strength and the anthropometric variables of the studied population.

Anthropometric Variables	Appendicular Skeletal Muscle Mass										Hand Grip Strength								
	Total			Women			Men				Total			Women			Men		
	n	r	*p*-Value	n	r	*p*-Value	n	r	*p*-Value		n	r	*p*-Value	n	r	*p*-Value	n	r	*p*-Value
Body mass (kg)	84	0.903	**<0.001**	38	0.949	**<0.001**	46	0.922	**<0.001**		84	0.319	**0.003**	38	0.468	**0.003**	46	0.109	0.472
NH Weight	84	0.882	**<0.001**	38	0.950	**<0.001**	46	0.897	**<0.001**		84	0.315	**0.004**	38	0.449	**0.005**	46	0.147	0.330
Height (cm)	84	0.694	**<0.001**	38	0.640	**<0.001**	46	0.421	**0.004**		84	0.666	**<0.001**	38	0.548	**<0.001**	46	0.452	**0.002**
BMI (kg/m^2^)	84	0.701	**<0.001**	38	0.879	**<0.001**	46	0.826	**<0.001**		84	0.048	0.666	38	0.352	**0.030**	46	−0.036	0.814
LTM (kg)	84	0.664	**<0.001**	38	0.505	**0.001**	46	0.594	**<0.001**		84	0.490	**<0.001**	38	0.212	0.200	46	0.348	**0.018**
LTI	84	0.433	**<0.001**	38	0.273	0.097	46	0.500	**<0.001**		84	0.238	**0.029**	38	0.007	0.968	46	0.221	0.141
Fat (kg)	84	0.614	**<0.001**	38	0.799	**<0.001**	46	0.653	**<0.001**		84	0.103	0.350	38	0.392	**0.015**	46	0.013	0.932
RFM	84	0.090	0.415	38	0.344	**0.034**	46	0.227	0.129		84	−0.146	0.185	38	0.196	0.238	46	−0.080	0.596
FTI	84	0.449	**<0.001**	38	0.723	**<0.001**	46	0.593	**<0.001**		84	−0.058	0.599	38	0.319	0.051	46	−0.063	0.677
ASM (BIS) (kg)											84	0.501	**<0.001**	38	0.531	**0.001**	46	0.188	0.210

NH weight, normally hydrated weight; BMI, body mass index; LTM, lean tissue mass; LTI, lean tissue index; RFM, relative fat mass; FTI, fat tissue index; ASM (BIS), appendicular skeletal muscle mass measured with bioimpedance spectroscopy; *p*-values < 0.05 are marked in bold; r, Pearson correlation coefficient.

**Table 4:** The associations between appendicular skeletal muscle mass and hand grip strength and the clinical variables of the studied population.

Clinical Variables	Appendicular Skeletal Muscle Mass									Hand Grip Strength								
	Total			Women			Men			Total			Women			Men		
	n	Mean ± SD	*p*-Value	n	Mean ± SD	*p*-Value	n	Mean ± SD	*p*-Value	n	Mean ± SD	*p*-Value	n	Mean ± SD	*p*-Value	n	Mean ± SD	*p*-Value
Serum albumin (g/dL)																		
<3.9	9	22.6 ± 5.4	0.073	3	23.2 ± 6.8	**0.011**	6	22.3 ± 5.2	0.896	9	23.6 ± 5.5	0.603	3	20.3 ± 1.2	0.642	6	25.3 ± 6.23	0.185
3.9–4.9	72	19.4 ± 4.8		33	16.4 ± 4.0		39	22.0 ± 3.9		72	25.5 ± 10.6		33	18.8 ± 5.7		39	31.2 ± 10.4	
Hemoglobin (g/dL)																		
<11.0	16	19.0 ± 5.8	0.580	8	17.6 ± 6.1	0.605	8	20.4 ± 5.6	0.221	16	23.3 ± 7.2	0.413	8	21.0 ± 6.3	0.209	8	25.7 ± 7.66	0.125
11.0–18.0	66	19.8 ± 4.7		30	16.7 ± 4.0		36	22.3 ± 3.6		66	25.7 ± 10.7		30	18.3 ± 5.0		36	31.8 ± 10.4	
eGFR (mL/min/1.73 m^2^)																		
≤29	62	19.4 ± 4.8	0.304	28	16.7 ± 4.5	0.694	34	21.6 ± 3.9	0.185	62	24.3 ± 9.5	0.131	28	18.2 ± 5.4	0.212	34	29.3 ± 9.30	0.146
30–44	22	20.6 ± 5.2		10	17.4 ± 4.5		12	23.4 ± 4.1		22	28.1 ± 11.4		10	20.7 ± 5.0		12	34.2 ± 11.7	
Serum creatinine (xULN)																		
<1.5	18	17.9 ± 4.6	0.138	15	17.0 ± 3.8	0.971	3	22.3 ± 6.3	0.191	18	21.3 ± 6.3	0.105	15	19.5 ± 4.9	0.786	3	30.3 ± 4.5	0.741
1.5–2.0	31	19.4 ± 5.1		14	17.2 ± 5.6		17	21.3 ± 4.0		31	24.4 ± 11.5		14	17.6 ± 4.6		17	29.9 ± 12.6	
2.0–3.0	27	20.5 ± 4.0		6	16.1 ± 1.7		21	21.8 ± 3.6		27	27.6 ± 9.3		6	19.3 ± 6.0		21	30.0 ± 8.7	
>3.0	8	22.2 ± 6.4		3	16.6 ± 6.5		5	25.6 ± 3.4		8	29.8 ± 11.7		3	20.5 ± 10.3		5	35.4 ± 9.1	
Serum urea (xULN)																		
≤1	3	24.0 ± 2.8	0.408	0	-	0.706	3	24.0 ± 2.8	0.833	3	50.3 ± 8.1	**<0.001**	0	-	0.937	3	50.3 ± 8.1	**0.003**
1–1.5	20	19.4 ± 5.1		11	16.5 ± 3.1		9	23.0 ± 4.8		20	23.2 ± 7.4		11	20.0 ± 4.9		9	27.1 ± 8.4	
1.5–2	22	19.1 ± 5.5		15	17.7 ± 5.3		7	21.9 ± 5.1		22	23.8 ± 10.9		15	18.8 ± 6.5		7	34.6 ± 10.7	
2–2.5	16	21.2 ± 3.7		3	19.7 ± 5.7		13	21.5 ± 3.3		16	25.9 ± 8.9		3	18.0 ± 1.0		13	27.7 ± 9.0	
>2.5	11	20.6 ± 5.5		4	16.4 ± 5.0		7	23.0 ± 4.3		11	24.7 ± 8.0		4	19.0 ± 7.0		7	28.0 ± 7.0	
Serum uric acid (mg/dL)																		
W: 2.4–5.7; M: 3.4–7.0	29	19.7 ± 5.2	0.965	9	15.8 ± 5.7	0.395	20	21.5 ± 3.9	0.263	29	28.2 ± 11.2	**0.039**	9	18.9 ± 5.7	0.937	20	32.4 ± 10.5	0.266
W: >5.7; M: >7.0	48	19.8 ± 5.1		27	17.3 ± 4.1		21	23.0 ± 4.4		48	23.3 ± 9.1		27	19.1 ± 5.3		21	28.8 ± 10.2	
OH (L)																		
<−1.0	9	25.0 ± 3.7	**<0.001**	4	23.3 ± 4.9	**0.006**	5	26.4 ± 2.1	**0.026**	9	29.6 ± 11.4	0.121	4	20.5 ± 7.9	0.715	5	36.9 ± 7.9	0.298
−1.0–1.0	47	18.1 ± 4.6		28	16.1 ± 3.8		19	21.1 ± 4.1		47	23.4 ± 10.1		28	18.5 ± 4.8		19	30.6 ± 11.6	
>1.0	28	20.7 ± 4.3		6	16.4 ± 4.1		22	21.8 ± 3.6		28	27.1 ± 9.2		6	19.8 ± 6.7		22	29.1 ± 8.8	
ROH (%)																		
<−7.0	8	24.7 ± 3.9	**0.003**	4	23.3 ± 4.9	**0.005**	4	26.2 ± 2.4	0.086	8	28.2 ± 11.3	0.536	4	20.5 ± 7.9	0.695	4	35.9 ± 8.7	0.374
−7.0–7.0	52	18.7 ± 4.7		29	16.4 ± 4.0		23	21.6 ± 4.0		52	24.4 ± 10.2		29	18.9 ± 5.3		23	31.3 ± 10.8	
>7.0	24	20.3 ± 4.5		5	14.9 ± 2.0		19	21.7 ± 3.9		24	26.2 ± 9.6		5	17.4 ± 3.9		19	28.5 ± 9.4	
Diabetes mellitus type 2																		
No	63	19.2 ± 4.4	0.118	28	16.7 ± 4.1	0.674	35	21.3 ± 3.5	**0.014**	63	26.0 ± 10.5	0.288	28	19.4 ± 5.6	0.295	35	31.2 ± 10.7	0.465
Yes	21	21.2 ± 6.1		10	17.4 ± 5.4		11	24.6 ± 4.6		21	23.2 ± 8.6		10	17.4 ± 4.2		11	28.6 ± 8.1	

eGFR, estimated glomerular filtration rate; ULN, upper limit of normal; W, women; M, men; OH, overhydration; ROH, relative overhydration; *p*-values < 0.05 are marked in bold.

**Table 5:** Stepwise forward multiple linear regression of hand grip strength in the studied population.

Independent Variables	Coefficient	95%CI	*p*-Value
Height (cm)	0.405	0.122; 0.688	**0.006**
Age (years)	−0.231	−0.353; −0.109	**<0.001**
Gender (men)	8.981	3.960; 14.002	**0.001**
Uric acid (mg/dL)	−1.078	−2.110; −0.045	**0.041**
OH (L)	−0.805	−1.579; −0.032	**0.042**

OH, overhydration; *p*-values < 0.05 are marked in bold.

## Data Availability

The data presented in this study are available upon request from the corresponding author. The data are not publicly available due to the Polish General Data Protection Regulation.

## Abbreviations

ASM	appendicular skeletal muscle mass
ATM	adipose tissue mass
BCM	body composition monitor
BIA	bioelectrical impedance analysis
BIS	multifrequency bioimpedance spectroscopy
BMI	body mass index
CKD	chronic kidney disease
CT	computed tomography
DXA	dual-energy X-ray absorptiometry
ECW	extracellular water
eGFR	estimated glomerular filtration rate
EWGSOP2	European Working Group on Sarcopenia in Older People
FFM	fat-free mass
FM	fat mass
FTI	fat tissue index
HGS	hand grip strength
ICW	intracellular water
LTI	lean tissue index
LTM	lean tissue mass
MDRD	modification of diet in renal disease formula
MRI	magnetic resonance imaging
NH weight	normally hydrated weight
OGTT	oral glucose tolerance test
OH	overhydration
PEW	protein-energy wasting
Rel Fat	relative fat
Rel OH	relative overhydration
SD	standard deviations
TBW	total body water
UA	uric acid
ULN	upper limit of normal
